# Socio-demographic characteristics and co-occurrence of depressive symptoms with chronic diseases among older adults in China: the China longitudinal ageing social survey

**DOI:** 10.1186/s12888-019-2305-2

**Published:** 2019-10-23

**Authors:** Zhenjie Wang, Hanmo Yang, Zhanyuan Guo, Bei Liu, Shen Geng

**Affiliations:** 1 Institute of Population Research, Peking University, Beijing, 100871 People’s Republic of China; 2 National School of Development, Peking University, Beijing, 100871 People’s Republic of China

**Keywords:** China, Elder population, Depressive symptoms, Chronic diseases

## Abstract

**Background:**

The aim of the current study is to assess the cross-sectional association of chronic non-communicable diseases (hypertension, diabetes mellitus, arthritis, and cerebrovascular) with depressive symptoms among older adults in China.

**Methods:**

Data was obtained from the China Longitudinal Ageing Social Survey (CLASS) conducted in 2014. A total of 7505 participants were included. Depressive symptoms status was assessed by 9-item Center for Epidemiological Studies Depression Scale (CES-D) Associations between depressive symptoms and chronic diseases, adjusting for so, demographics and chronic diseases risk factors were assessed by using logistic regression model.

**Results:**

We found negative associations between depressive symptoms and several socioeconomic factors, including education attainment and economic level. Widowed/divorced/ unmarried individuals are more likely to suffer from depressive symptoms. Hypertension (Odds ratio:1.29 [95%CI:1.16, 1.42]), diabetes (1.41 [95%CI:1.19,1.67]), arthritis (1.72 [1.52, 1.96]), and cerebrovascular disease (1.69 [1.41, 2.02]) were found to be associated with depressive symptoms.

**Conclusions:**

Most depressive symptoms cases were found to be significantly associated with chronic diseases. Our findings have provided evidence for understanding co-morbid depressive symptoms with chronic diseases, which could help clinicians to evaluate, diagnose and manage depression promptly.

## Background

Depression, which affects approximately 350 million people across the world, is an important public health issue [1, 2]. Depression had become the fourth leading cause of disease burden [1] and will become the second leading cause of disease burden in near future [3]. People with depression are usually associated with low quality of life, prevalence of cancer, chronic diseases, and higher mortality [4]. Depression causes a heavy burden on families, communities and health services in both high-income and low- and middle-income countries [5, 6].

In China, the prevalence of depression had aggressively increased to 17% among the elderly population [7, 8]. A number of studies suggested that there was an inverse or U-shaped association between prevalence of depression and age [9–11]. Moreover, several socioeconomic variables are related to depression [12], although these associations are inconsistent [8, 13]. Many studies found that low education level is associated with a high risk of depression in China [14, 15].

Several chronic diseases were found to be associated with depression [16–18]. With age increases, the presence of biological risk factors makes elder people vulnerable to depression [19, 20]. The positive correlation between depression and specified chronic diseases were found among the adult population in the developed countries [19, 20]. However, limited studies have found such a correlation in the developing countries [21–23], although the development of a chronic disease can bring on depression reaction. Because the chronic diseases and depression shared similar risk factors and pathophysiological process, the association between chronic diseases and depression have presented strong link [19, 20].

Currently, there is limited epidemiologic evidence linking chronic diseases status with depression risk among Chinese older population. Therefore, we investigated the association of chronic diseases status with depressive symptoms risk using data from the Chinese Longitudinal Ageing Social Survey (CLASS) in China.

## Methods

### Setting and sample

In the current study, we used the data from the baseline of the Chinese Longitudinal Ageing Social Survey (CLASS) that was collected by the National Survey Research Center, Renmin University of China. This survey was stratified, multi-stage, probabilistic sampling survey, which coved 28 provincial areas in China. Details of the design and conduct of the study have been described elsewhere [24]. A total of 11,511elderly aged 60 or above was interviewed by face to face. In the present study, the sample is comprised of 7505 subjects aged 60 or above who had finished the Center for Epidemiological Studies Depression Scale (CES-D) and other independent variables of interest.

### Depressive symptoms assessment

Depressive symptoms was assessed using an abbreviated nine-item Center for Epidemiological Studies Depression Scale (CES-D), which is reliable and valid for detecting depressive symptoms among Chinese older adults [25]. In the CLASS study, the internal Cronbach’s alpha for CES-D used was 0.75. Using Kohout’s formula [26], standardized cut scores are determined by dividing the total possible score on a short CES-D scale by 60 (the total possible score on the full 20-item CES-D) and multiplying that number by 16 (the established cut score on the full 20-item CES-D. For current study, on a 9-item scale, the total possible score is 18 (9 items multiplied by 2, the highest response). That total score is divided by 60, which equals 0.3. Then, the 0.3 is multiplied by 16, resulting in a standardized cut score of 4.8 for the 9-item form of the CES-D.

### Independent variable of interest

We included the following socioeconomic characteristics in our study: age (60–64, 65–69, 70–74, 75–79, 80+), gender (male, female), residence (rural, urban), marital status (married, widowed/divorced/unmarried), education level (junior high school and above, primary school, never attended school), ethnicity (Han, Others), and living arrangements (lives alone, lives with others). Income is categorized into five levels using the quintiles of household income (Yuan) (Q1: ≤ 3000, Q2: > 3000 and ≤ 10,000, Q3: > 10,000 and ≤ 24,000, Q4: > 24,000 and ≤ 36,000, Q5: > 36,000). We dichotomized the physical disability status, which was assessed by using ten-item version of the activities of daily living (ADL) scale, into two groups (“no functional problems”= 0, “has at least one limitation”=1) [27]. The participants were also asked whether they had one of the following health problems (covered 23 chronic disease), including hypertension (Yes, No), diabetes mellitus (Yes, No), arthritis (Yes, No), cerebrovascular disease (Yes, No), liver disease, and so on. Number of comorbid chronic disease were further categorized into “0”, “1” and “≥2”.

### Statistical analysis

The difference between subjects with/without depressive symptoms was tested by χ^2^ test with proportions. The logistic regression model was used to calculate the adjusted odds ratios (OR) and 95% confidence interval (CI) of depressive symptoms (yes/no) with the covariates: age group, gender, residence, marital status, education, ethnicity, physical disability, living arrangement, and wealth quantiles. Separate multiple logistic regression models were used to assess the association of depressive symptoms with each chronic disease. In each model, the exposure of interest was one chronic disease and other covariates (mentioned above) were included. All two-way interactions between each chronic disease and covariates were assessed in multivariable adjusted models. The trend of the association was assessed with ordinal scores assigned to number of comorbid chronic disease. Statistical significance was declared with a two-sided *p*-value < 0.05. Statistical analyses were performed using SAS version 9.3 (SAS Institute Inc., Cary, NC, USA).

## Results

Selected characteristics of the subjects with/without depressive symptoms are summarized in Table 1. In the current study, male subjects, urban residents, people living with others, and the Han nationality accounted for a majority of the Chinese elderly population. As compared with the subjects without depressive symptoms, the depressive symptoms cases were slightly older and had a higher proportion of female subjects and physical disability, but a lower proportion of them are living with others.

**Table 1:** Subjects’ characteristics by their depressive symptoms status according to the nine-item Center for Epidemiological Studies Depression Scale (CES-D) among elder population in China ^a^

Characteristics	With depressive symptoms	Without depressive symptoms	*P*
Age groups (years)			
60–64	1070 (33.5)	1605 (37.2)	< 0.001
65–69	704 (22.1)	989 (22.9)	
70–74	547 (17.2)	769 (17.8)	
75–79	472 (14.80)	530 (12.3)	
80+	397 (12.5)	422 (9.8)	
Gender			
Male	1627 (51.0)	2427 (56.2)	< 0.001
Female	1563 (49.0)	1888 (43.8)	
Residence			
Rural	1316 (41.2)	1208 (28.0)	< 0.001
Urban	1874 (58.8)	3107 (72.0)	
Marital status			
Married	2039 (63.9)	3318 (76.9)	< 0.001
Widowed/divorced/ Unmarried	1151 (36.1)	997 (23.1)	
Education level			
Never attended school	816 (25.6)	651 (15.1)	< 0.001
Primary school	1268 (39.8)	1411 (32.7)	
Junior High school and above	1106 (34.7)	2253 (52.2)	
Ethnicity			
Han	2993 (93.8)	4080 (94.6)	0.19
Others	197 (6.2)	235 (5.4)	
Living arrangement			
Live with others	2661 (83.4)	3921 (90.9)	< 0.001
Live alone	529 (16.6)	384 (9.1)	
Physical disability			
No function problems	2754 (86.3)	4112 (95.3)	< 0.001
One and more functioning limitations	436 (13.7)	203 (4.7)	
Wealth quantile			
Q1 (lowest)	926 (29.0)	692 (16.0)	< 0.001
Q2	718 (22.5)	694 (16.1)	
Q3	705 (22.1)	1067 (24.7)	
Q4	520 (16.3)	1003 (23.2)	
Q5 (highest)	321 (10.1)	859 (19.9)	
Hypertension			
No	2011 (63.0)	2948 (68.3)	< 0.001
Yes	1179 (37.0)	1367 (31.7)	
Diabetes mellitus			
No	2863 (89.7)	3950 (91.5)	0.009
Yes	327 (10.3)	365 (8.5)	
Arthritis			
No	2450 (76.8)	3782 (87.7)	< 0.001
Yes	740 (23.2)	533 (12.3)	
Cerebrovascular disease ^b^			
No	2842 (89.1)	4058 (94.0)	< 0.001
Yes	348 (10.9)	257 (6.0)	
Number of comorbid chronic disease			
0	553 (17.3)	1467 (34.0)	< 0.001
1	917 (28.8)	1307 (30.3)	
≥ 2	1720 (53.9)	1541 (35.7)	

^a^ The difference between subjects with/without depressive symptoms was tested by χ^2^ test

^b^ Including stroke

The associations between subjects’ characteristics and depressive symptoms were presented in Table 2. There was a significantly weaker association between residence, education, and wealth quantile with depressive symptoms. People who were widowed/divorced/ unmarried, with physical disability or live alone were 1.34 times (*p*-values< 0.001), 2.82 times (*p*-values< 0.001), and 1.62 tines (*p*-values = 0.017) as likely to have depressive symptoms, respectively.

**Table 2:** Odds ratio (95% confidence interval) of depressive symptoms risk according to characteristics

	Reference		OR (95% CI)
Age group (years)	60–64	65–69	0.95 (0.83–1.08)
		70–74	0.91 (0.79–1.05)
		75–79	1.05 (0.90–1.24)
		80+	0.96 (0.80–1.15)
Gender	Male	Female	1.01 (0.91–1.12)
Residence	Rural	Urban	0.87 (0.77–0.98)
Marital status	Married	Widowed/divorced/ unmarried	1.34 (1.18–1.53)
Education	Never attended school	Primary school	0.90 (0.79–1.04)
		Junior high school and above	0.70 (0.60–0.81)
Ethnicity	Han	Others	1.01 (0.82–1.24)
Physical disability	No function problems	One and more functioning limitations	2.82 (2.35–3.39)
Living arrangement	Living with others	Living alone	1.62 (1.48–1.92)
Wealth quantile	Q1 (lowest)	Q2	0.84 (0.72–0.97)
		Q3	0.61 (0.52–0.71)
		Q4	0.53 (0.45–0.63)
		Q5 (highest)	0.41 (0.33–0.49)

Abbreviations: *CI* confidence interval, *OR* odds ratio

The odds ratio of chronic diseases for depressive symptoms was presented in the Table 3. After controlling for most important confounders, having hypertension [OR:1.29, 95%CI: (1.16–1.42)], diabetes mellitus [OR:1.41, 95%CI: (1.19–1.67)], arthritis [OR: 1.72, 95%CI: (1.52–1.96)], and cerebrovascular disease [OR: 1.69, 95%CI: (1.41–2.02)] were associated with depressive symptoms (all *p*-values< 0.001). The odds ratios of depressive symptoms presented positive associated with the number of chronic disease (*P*_trend_ <  0.001).

**Table 3:** Model adjusted association between depressive symptoms and chronic diseases controlled for socioeconomic factors

	COR (95% CI)	AOR (95% CI)^a^
Hypertension		
No	Reference	Reference
Yes	1.28 (1.15–1.39)	1.29 (1.16–1.42)
Diabetes mellitus		
No	Reference	Reference
Yes	1.24 (1.06–1.45)	1.41 (1.19–1.67)
Arthritis		
No	Reference	Reference
Yes	2.14 (1.90–2.42)	1.72 (1.52–1.96)
Cerebrovascular disease ^b^		
No	Reference	Reference
Yes	1.93 (1.63–2.29)	1.69 (1.41–2.02)
Number of comorbid chronic disease		
0	Reference	Reference
1	1.86 (1.70–2.12)	1.82 (1.59–2.08)
≥ 2	2.96 (2.63–3.34)	2.57 (2.27–2.92)
*P*_*trend*_	< 0.001	< 0.001

Abbreviations: *COR* crude odd ratio, *AOR* adjusted odds ratio

^a^ Adjusted for Age group, gender, residence, marital status, education, ethnicity, physical disability, living arrangement, and wealth quantiles

^b^ Including stroke

## Discussions

Very limited studies had explored the association between depressive symptoms and chronic diseases in China. More than 80% of subjects who were with depressive symptoms had chronic diseases. Our findings showed a strong positive association between depressive symptoms and chronic diseases under consideration of socioeconomic confounders.

We found significant associations between depressive symptoms and several socioeconomic factors, such as education attainment, economic level, physical disability and so on. In previous studies, positive associations between gender, age and depressive symptoms were reported [28]. However, we did not observed similar association in the current study. Similar positive association between depressive symptoms and being without spouse or having a lower education level or less wealth was also observed [28]. Low socioeconomic status could make people more likely with depressive symptoms through some mechanisms such as unsafe and unstable environments, malnutrition, reduced possibility of education, and limitedly access to health care services [28].

In the current study, we observed increased odds ratios of depressive symptoms in those with chronic disease, including diabetes mellitus and cerebrovascular disease. The associations between diabetes, hypertension with depressive symptoms were similarly compared with previous studies [19, 20]. These associations could be explained by several mechanisms, including unhealthy lifestyle, shared genetic influences, and inflammation in both diseases [29]. Previous studies also suggested some behavioral or biological pathways that link depressive symptoms with diabetes [30]. Furthermore, we found a positive association between depressive symptoms and arthritis after taking socioeconomic factors into consideration. The risk of depressive symptoms of participants with arthritis is 1.72 times as much as those without arthritis. Previous studies suggested that pain and physical disability experience could lead a side effect on their mood [20]. Another explanation is subjects having depressive symptoms might be more sedentary than those without depressive symptoms, which could cause an increased bone resorption and a lowered bone formation [31, 32].

Our study has several strengths, including the large sample size, the population-based design, and adjustment for a wide range of socioeconomic characteristics. Another noticeable strength is that the measure of all physical illnesses took place prior to the CES-D measurement, thus it helped to minimize the risk of reverse causation. However, our study also has several limitations, which should be taken into consideration by future researchers. As a cross-sectional study, we cannot deduce causative pathways between depressive symptoms and chronic diseases; therefore, future longitudinal research is needed to assess the causal relationships between depressive symptoms, chronic diseases and socioeconomic factors. Moreover, the CLASS does not provide sufficient information on lifestyle factors (i.e., weight, height, smoking, alcohol drinking, and so on) as risk factor of depressive symptoms, which should be cautioned in future studies.

## Conclusions

In the current study, our findings suggest a particular need for recognition of depressive symptoms in the primary care. The increasing population with chronic non-communicable and mental health should be cautioned for public health policy makers and future studies. Our findings have provided evidence for understanding co-morbid depressive symptoms with chronic diseases, which could help clinicians to evaluate, diagnose and manage depression promptly.

## Data Availability

The data is available on request from Institute of Gerontology and National Survey Research Center at Renmin University of China, who can be contacted through http://class.ruc.edu.cn/index.php?r=index/index&hl=en. Shen Geng had received permission and accessed to all the data.

## Abbreviations

CES-D: Center for Epidemiological Studies Depression Scale
CLASS: the China Longitudinal Ageing Social Survey
